# Chemical Defence in a Millipede: Evaluation and Characterization of Antimicrobial Activity of the Defensive Secretion from *Pachyiulus hungaricus* (Karsch, 1881) (Diplopoda, Julida, Julidae)

**DOI:** 10.1371/journal.pone.0167249

**Published:** 2016-12-01

**Authors:** Slaviša Stanković, Ivica Dimkić, Ljubodrag Vujisić, Sofija Pavković-Lučić, Zvezdana Jovanović, Tatjana Stević, Ivana Sofrenić, Bojan Mitić, Vladimir Tomić

**Affiliations:** 1 Department of Microbiology, University of Belgrade – Faculty of Biology, Belgrade, Serbia; 2 Department of Organic Chemistry, University of Belgrade – Faculty of Chemistry, Belgrade, Serbia; 3 Department of Genetics and Evolution, University of Belgrade – Faculty of Biology, Belgrade, Serbia; 4 Department of Animal Development, University of Belgrade – Faculty of Biology, Belgrade, Serbia; 5 Institute for Medicinal Plants Research “Dr. Josif Pančić”, Belgrade, Serbia; University of Padova, Medical School, ITALY

## Abstract

The chemical defence of the millipede *Pachyiulus hungaricus* is reported in the present paper, in which a chemical characterization is given and antimicrobial activity is determined. In total, independently of sex, 44 compounds were identified. All compounds belong to two groups: quinones and pentyl and hexyl esters of long-chain fatty acids. The relative abundances of quinones and non-quinones were 94.7% vs. 5.3% (males) and 87.3% vs. 12.7% (females), respectively. The two dominant quinones in both sexes were 2-methyl-1,4,-benzoquinone and 2-methoxy-3-methyl-1,4-benzoquinone. Antibacterial and antifungal activity of the defensive secretion was evaluated *in vitro* against seven bacterial strains and eight fungal species. With the aid of a dilution technique, the antimicrobial potential of the secretion and high sensitivity of all tested strains were confirmed. The lowest minimum concentrations of these compounds (0.20–0.25 mg/mL) were sufficient for inhibition of *Aeromonas hydrophila*, *Listeria monocytogenes* and Methicillin resistant *Staphylococcus aureus* (MRSA). The growth of eight tested fungal species was inhibited by slightly lower concentrations of the secretion, with *Fusarium equiseti* as the most sensitive fungus and *Aspergillus flavus* as the most resistant. Values of MIC and MFC in the employed microdilution assay ranged from 0.10 to above 0.35 mg/mL. The given extract contains antimicrobial components potentially useful as therapeutic agents in the pharmaceutical and agricultural industries.

## Introduction

Millipedes are the third most diverse class of terrestrial arthropods, with about 12 000 formally described species [1]. One of the most conspicuous features of the numerous members of the class Diplopoda is the presence of a pair of exocrine defence glands in the body somites. These glands produce a variety of volatile compounds provisionally grouped into alkaloids, quinones, phenols and cyanogenic compounds [2–8]. Species with chemical defence have a pair of ozadenes in most of the pleurotegites, opening laterally or dorsally via ozopores. Millipedes are known to release the content of these glands mainly during defensive behaviour, although other functions have been hypothesized for it, such as being an alarm signal, a substance containing pheromones, a repellent against parasites or a carrier of antibacterial and antifungal activity [9].

However, the chemical defence sequester of these animals has been analysed in only 170 species [3–8,10]. The millipede family Julidae comprises more than 600 species from the western part of the Palaearctic region [1]. As throughout the whole class, knowledge about the composition and functions of the defensive fluid in members of the family Julidae is scarce [10,11]. We here focus our attention on the species *Pachyiulus hungaricus* (Karsch, 1881), one of the largest and most robust of European millipedes (with body length of ca. 10–15 cm), belonging to the family Julidae and distributed mainly on the Balkan Peninsula. The species seems to be aposematic, since the body has red circular stripes on most of the pleurotegites. Individuals mainly inhabit litter and humus or sometimes take shelter under stones or roots of trees. There are no data concerning the defence secretion of any member of the genus *Pachyiulus*. To provide additional information about the defence sequester of the julids, we here give a chemical characterization of the defensive fluids of *P*. *hungaricus* and report for the first time the effects of a secretion extract obtained from one member of the order Julida on some pathogenic organisms. With emergence of new and reemergence of old infectious diseases, urgent need for new antimicrobial agents arises, especially concerning the increase of antimicrobial multiple drug resistance microorganisms around the world. The present study was aimed at investigating the *in vitro* antimicrobial activity of defensive secretions on some important bacterial and fungal strains.

## Material and Methods

### Collection and handling of millipedes

Millipedes were collected during May of 2013 on Mt. Avala (44.6892° N, 20.5161° E), near Belgrade, Serbia. The millipedes were stored in plastic boxes with a layer of decomposed litter, kept under laboratory conditions for few days at 10°C in the dark and sprayed with water every day. We confirmed that no specific permissions were required by authorities for the locations or activities involved. We also confirmed that the scope of our study did not involve endangered or protected species. All analyzed specimens are deposited in the Collection of the Institute of Zoology, University of Belgrade–Faculty of Biology (voucher numbers IZBJU 50–58).

### Collection of defensive secretions

Collection of secretions was accomplished using the mechanical stress approach. Male and female individuals were mechanically stressed in a closed test tube (20 × 150 mm). Collected secretions were used for chemical analyses and antimicrobial tests after dissolving in suitable solvents. To eliminate the effects of composition-altering oxidation and degradation of compounds, all secretions were subjected to GC-MS analysis and antimicrobial testing immediately after preparation.

### Chemical analyses

The GC and GC-MS analyses were performed on an Agilent 7890A GC system equipped with 5975C MSD and FID, using an HP-5 MSI capillary column (Agilent Technologies, 0.25 mm × 30 m I.D., 0.25 μm film thickness). Samples were injected using a splitless mode. The injection volume was 1 μL and injector temperature 250°C. The carrier gas (He) flow rate was 1.0 mL/min at 210°C (constant pressure mode). The column temperature was linearly programmed in a range of 60-315°C at a rate of 3°C/min with a final 15-min hold. The transfer line was heated at 315°C. The FID detector temperature was 300°C. Electron ionization (EI) mass spectra (70 eV) were acquired in the *m/z* range of 35–550, the ion source and quadrupole temperatures being 230 and 150°C, respectively. Chemical ionization (CI) mass spectra were obtained using isobutane (with energy of 235 eV) in the *m/z* range of 60–550, the ion source temperature being 300°C and quadrupole temperature 150°C [7,10].

A library search and mass spectral deconvolution and extraction were performed using MSD ChemStation data analysis software, ver. E.02.02, integrated with DRS (Deconvolution Reported Software) and NIST AMDIS (Automated Mass Spectral Deconvolution and Identification System) software, ver. 2.70. The EI MS search was performed with our own library, which contains 4,951 spectra, and the commercially available NIST11 and Wiley07 libraries, containing more than 500 000 EI mass spectra. We used CI MS data for identification and/or confirmation of molecular formula of compounds, and for determination of the alcoholic and acidic part of esters. Percentages (relative) of the identified compounds were computed from the corresponding GC-FID peak areas. Also, for most of the quinones we used published data in literature where these compounds were previously identified [7,10,12].

### Preparation of defensive extract

The extraction method used in this study was as follows: four females were irritated by stress until excretion of the defensive secretion from their glands was elicited. The secretion was dissolved in 10 mL of methanol. The extract was evaporated to a dry residue on an evaporator (Rotavapor R-215, Buchi, Switzerland), dissolved in 50% of methanol and used at a final concentration of 10 mg/mL in further tests.

### Pathogenic strains and culture conductions

Antibacterial activity of the defensive secretion from *P*. *hungaricus* was determined against seven bacterial strains (*Aeromonas hydrophila* ATCC 49140, *Pseudomonas aeruginosa* ATCC 15442, *Escherichia coli* ATCC 25922, *Staphylococcus aureus* ATCC 25923, Methicillin resistant *Staphylococcus aureus* (MRSA) ATCC 33591, *Listeria monocytogenes* 1/2a ATCC 19111, *Bacillus subtilis* ATCC 6633 and *Xanthomonas arboricola*) and eight species of fungi (*Aspergillus flavus*, *Aspergillus niger*, *Fusarium subglutinans*, *Fusarium semitectum*, *Fusarium equiseti*, *Penicillium* sp., *Gliocladium roseum* and *Chaetomium* sp.). The reference strains of bacteria used in this study were obtained from the American Type Culture Collection (ATCC) and from existing laboratory collections, except for the *Xanthomonas arboricola* strain, which was isolated from walnut fruit previously [13]. The bacterial strains were cultured in MHB and MHA (Müller-Hinton broth and agar, HiMedia, Mumbai, India), with the exception of *L*. *monocytogenes*, which was cultured in BHI (brain-heart infusion) broth (Biomedics, Madrid, Spain). Growth temperature was 37°C for all ATTC strains and 30°C for *X*. *arboricola*. The tested fungi were isolated and previously identified [14,15] from dried medicinal plant species that are mostly used in the manufacture of various products, especially teas. Samples were taken from the warehouse of the Dr. Josif Pančić Instutute of Medicinal Plant Research, Belgrade. Fungal isolates were cultured on potato dextrose agar (PDA) and on malt extract agar (MEA) at a growth temperature of 25°C.

### Antibacterial activity

Antibacterial assays were carried out by well-diffusion and microdilution methods to determine antibacterial activity of compounds against the tested pathogenic bacteria. The bacterial suspensions were adjusted to McFarland standard turbidity (0.5) (BioMérieux, Marcy-l'Étoile, France), which corresponds to 1×10^8^ CFU/mL. Inocula were prepared and stored at 4°C until use. All experiments were performed in triplicate.

#### Well-diffusion assay

A well-diffusion test [16] was used as a preliminary screening method in determining a defensive extract’s antimicrobial potential. Sterile molds for the wells (made from the bottom parts of 200-μL pipette tips, 5 mm in diameter) were placed on MHA/BHI, which was used as the solid medium, and MHB/BHI soft agar previously inoculated with 50 μL of the appropriate strain was uniformly spread on MHA/BHA. After the soft agar solidified, the molds were removed and 10 μL of the tested defensive extract was added to the wells. A measured volume (20 μL) of 50% methanol was used as a negative control. The petri dishes were incubated for 24 h at the optimal temperature for indicator strains. The level of bacterial susceptibility was determined according to zone diameter. Zones of inhibition were measured in mm.

#### Microdilution assay

A broth microdilution method was used to determine the minimum inhibitory concentration (MIC) and minimal bactericidal concentration (MBC) of the extract obtained from *P*. *hungaricus* against tested bacterial strains. A modified version of the method described by Sarker et al. [17] was used to determine MIC and MBC values. In total, twelve two-fold serial dilutions with MHB medium in 96-well microtitre plates were performed, except in the case of *L*. *monocytogenes*, for which BHI medium was used. The concentration of extract tested was in the range from 2–0.05 mg/mL. The final concentration of 50% methanol as a solvent was 10%. Besides a negative control, a sterility control and a control for the solvent (methanol), the antibiotic rifampicin (Sigma-Aldrich, Carlsbad, CA, USA) was also tested as a positive control. All dilutions were done in triplicate. Each well, except for the sterility control, was inoculated with 20 μL of bacterial culture (approx. 1×10^8^ CFU/mL), reaching a final volume of 200 μL. The higher inoculum of bacterial cultures was used due to the increased coloration of the extract. Lastly, 22 μL of resazurin (0.675 mg/mL) was added to each well. The plates were incubated for 24 h at the optimal temperature for indicator strains. All tests were performed in a lighted environment, but the plates were incubated in the dark. Resazurin is an oxidation-reduction indicator used for evaluation of cell growth. It is a blue non-fluorescent and non-toxic dye that becomes pink and fluorescent when reduced to resorufin by oxidoreductases inside viable cells [17]. The lowest concentration which showed no change in colour was defined as the MIC. The MBC was determined by sub-culturing the test dilutions from each well without colour change on agar plates and incubating for 18–24 h. The MBC is defined as the concentration at which 99.9% of killing the starting inoculum is observed. The results are expressed in mg/mL.

### Antifungal activity

Antifungal activity was tested using eight fungal strains isolated from medicinal drugs. A modified version of the microdilution technique described by Daouk et al. [18] was used to investigate antifungal activity of the defensive secretion from *P*. *hungaricus*. Fungal spores were washed from the surface of malt agar (MA) plates (malt extract, 50 g/L; agar, 15 g/L) with sterile 0.85% 113 saline containing 0.1% Tween 80 (*v/v*). The spore suspension was adjusted with sterile saline to a concentration of approximately 1.0 × 10^5^ in a final volume of 100 μL per well. The inocula were stored at 4°C for further use. Dilutions of the inocula were cultured on solid MA to verify the absence of contamination and to check validity of the inoculum. Determination of MIC values was performed by a microdilution technique using 96-well microtitre plates. Tryptic bile soy broth (TBS) (in g/L: casein, 20; bile salts, 1.5; X-B-D glucuronic acid, 0.075; dimethyl sulphoxide, 3) was used as the basis in the well, to which 0.01% Tween 80, different volumes of the investigated defensive extract and the fungal inoculum were added. The concentration of extract tested was in the range from 0.35–0.05 mg/mL. The microplates were incubated for 72 h at 28°C. The lowest concentrations without visible growth were defined as the minimal concentrations which completely inhibit fungal growth (MIC). The minimum fungicidal concentrations (MFC) were determined by serial subcultivation of a 2-μL volume on microtitre plates containing 100 μL of broth per well and further incubation for 72 h at 28°C. The lowest concentration with no visible growth was defined as the MFC, indicating 99.5% killing of the original inoculum compared to fluconazole, used as a positive control.

## Results and Discussion

### Chemical identification

**Fig 1:**
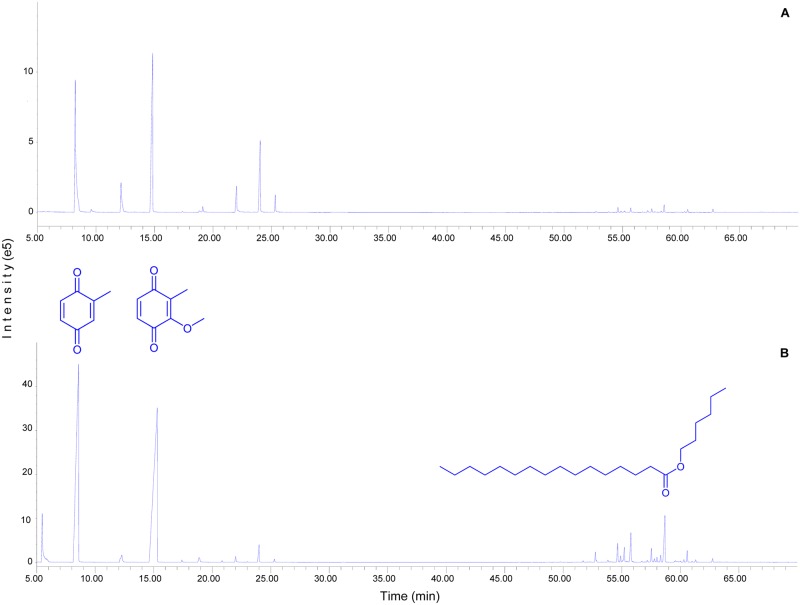
GC-FID profiles of A) male and B) female secretions of the millipede *Pachyiulus hungaricus* (Karsch, 1881) with chemical structure of most dominant quinone and non quinone compounds.

Analysis of the DCM extract of *P*. *hungaricus* secretions obtained by mechanical stress revealed the presence of 44 compounds [7,10,19] (Table 1) according to obtained GC-FID profiles (Fig 1).

**Table 1:** Chemical composition of the defensive secretion in *Pachyiulus hungaricus* (Karsch, 1881).

No.	Quinones	R. time (min)	RI measured/authentic reference**	Male (%)	Female (%)
1	1,4-Benzoquinone (*p*-benzoquinone)	5.47	920/920	0.1	4.0
2	2-Methyl-1,4-benzoquinone	8.58	1025/1025	30.2	36.8
3	2-Hydroxy-3-methyl-1,4-benzoquinone	12.28	1123/1129	6.5	0.9
4	2-Methoxy-3-methyl-1,4-benzoquinone	15.31	1193/1183	36.3	43.1
5	2-Methoxy-1,4-benzoquinone	17.41	1240/1240	0.1	0.1
6	1,4-Dihydroxy-benzene (hydroquinone)	18.87	1273	0.3	0.5
7	Not identified	19.12	1280	0.9	-
8	2,3-Dimethoxy-1,4-benzoquinone	20.84	1318/1320	-	0.1
9	2-Methyl-hydroquinone	22.00	1345/1341	4.6	0.4
10	2,3-Dimethoxy-hydroquinone	23.00	1368/1373	trace	trace
11	2-Methyl-3,4-methylene-dioxyphenol	24.01	1389/1390	13.4	1.2
12	2-Methoxy-3-methyl-hydroquinone	25.31	1420/1425	2.3	0.2
	Pentyl and Hexyl Esters*				
13	Pentyl tetradecanoate	49.75	2087/2100	trace	trace
14	Hexyl tetradecanoate^a^	51.59	2149	trace	trace
15	Pentyl pentadecanoate^b^	51.70	2153	trace	0.1
16	Pentyl pentadecanoate^b^	51.96	2162	trace	trace
17	Hexyl pentadecenoate	52.07	2165	trace	trace
18	Hexyl tetradecanoate^a^	52.75	2187/2162	0.1	0.6
19	Pentyl pentadecanoate^b^	52.83	2191	trace	0.1
20	Hexyl pentadecanoate^c^	53.82	2223	0.1	0.1
21	Hexyl pentadecanoate^c^	54.65	2251	0.6	1.2
22	Hexyl pentadecanoate^c^	54.90	2260/2262	0.2	0.3
23	Hexyl pentadecanoate^c^	55.09	2266	trace	0.1
24	Not identified	55.21	2270	0.2	0.8
25	Hexyl pentadecanoate^c^	55.78	2289	0.6	2.1
26	Hexyl hexadecanoate^d^	55.95	2294	0.1	0.1
27	Hexyl hexadecenoate^e^	56.71	2320	0.1	0.1
28	Not identified	57.18	2336	0.3	0.1
29	Hexyl hexadecanoate^d^	57.54	2348	0.5	0.8
30	Hexyl hexadecanoate^d^	57.78	2356	0.1	0.2
31	Hexyl hexadecenoate^e^	58.01	2363	trace	0.3
32	Hexyl hexadecenoate^e^	58.32	2374	0.2	0.4
33	Hexyl hexadecanoate^d^	58.69	2386/2381	1.0	3.9
34	Hexyl heptadecanoate^f^	59.52	2418	trace	trace
35	Hexyl heptadecanoate^f^	59.59	2418	trace	0.1
36	Hexyl heptadecenoate^g^	59.79	2426	trace	trace
37	Hexyl heptadecenoate^g^	60.02	2434	trace	0.1
38	Hexyl heptadecanoate^f^	60.31	2445	0.1	0.1
39	Hexyl heptadecanoate^f^	60.60	2456	0.4	0.7
40	Hexyl heptadecanoate^f^	61.01	2472/2468	trace	trace
41	Hexyl heptadecanoate^f^	61.32	2482	trace	0.1
42	Not identified	62.77	2534	0.5	0.2
43	Hexyl octadecanoate	63.96	2582/2564	trace	trace
44	Not identified	66.85	2685	0.1	trace
	Sum of identified			98	98.9

^a^ Isomer compound of Hexyl tetradecanoate,

^b^ Isomer compound of Pentyl pentadecanoate,

^c^ Isomer compound of Hexyl pentadecanoate,

^d^ Isomer compound of Hexyl hexadecanoate,

^e^ Isomer compound of Hexyl hexadecenoate,

^f^ Isomer compound of Hexyl heptadecanoate,

^g^ Isomer compound of Hexyl heptadecenoate.

*Retention index of linear n-pentyl and n-hexyl esters appropriate acids. Other compounds are bifurcated isomers of these esters.

**RI measured/authentic reference [7,10,19].

Unfortunately, five compounds (**7**, **24**, **28**, **42** and **44**) present in 2% of male secretions and 1.1% of female secretions were not identified. Generally, all compounds belong to two groups: quinones (compounds **1**–**12**); and pentyl and hexyl esters of saturated and unsaturated fatty acids with chain lengths ranging from C_14_ to C_18_ (compounds **13**–**44**).

Two quinones are dominant, viz., compounds **2** and **4**, with relative abundance of 66.5% (males) and 79.9% (females). All others quinones represent minor compounds of defensive fluids (Table 1). One exception is compound **11** in males, with 13.4% relative abundance, but it is known that compound **11** is a product of the cyclodehydrogenation of 2-methoxy-3-methyl-1,4-benzoquinone (compound **4**) [20]. Most of the quinones detected in this study were known from different previously analysed members of the family Julidae [7,10]. Hydroquinone is here detected for only the second time in the whole family Julidae [11]. Compound **4** is the only quinone detected in all analysed members of the order Julida [one exception is the parajulid *Uroblaniulus canadensis* (Newport, 1844); this is possibly a result of low sensitivity of the equipment used by Weatherston and Percy [21] to characterize the chemical composition of its defensive fluids]. It also appears in many members of the orders Spirobolida and Spirostreptida, but its presence is not universal in these orders [22]. 2-Methyl-1,4-benzoquinone (**2**) is common in juliform millipedes (Spirobolida, Spirostreptida, and Julida), but its appearance is likewise not universal in these millipedes [12,23]. Furthermore, the composition of other minor quinones in *P*. *hungaricus*, as in julids in general, showed a species-specific pattern. In other words, all analysed members of the order Julida have a specific quinonic chemoprofile, especially regarding minor compounds; there are no identical julid quinonic chemoprofiles [7,11,12]. Moreover, we detected for the first time some minor sex-related differences in the composition of quinones (Table 1).

Interestingly, in this study we identified a huge number of esters in defensive fluids of *P*. *hungaricus* (Table 1), the functional role of which will be a subject of further study. In order to confirm that these compounds are really part of defensive fluids (not cuticular components), we devised an experiment entailing dissection of a few first segments without ozadenes and washing of them with dichloro methane. In that cuticular wax secretion, long-chain esters were absent. The relative abundances of quinones and non-quinones (esters) in the defensive fluids of *P*. *hungaricus* obtained by means of mechanical stress were 94.7% vs. 5.3% (males) and 87.3% vs. 12.7% (females). All esters were tentatively identified as isomers of 10 different esters (Table 1). The most abundant saturated esters in both males and females were hexyl pentadecanoate and hexyl hexadecanoate, while the most abundant unsaturated ester was hexyl hexadecenoate. A previous report suggested that some compounds of this kind may be important for effectiveness of quinones, but also as possible informative molecules necessary for communication in these arthropods [9].

### Chemical defence

**Fig 2:**
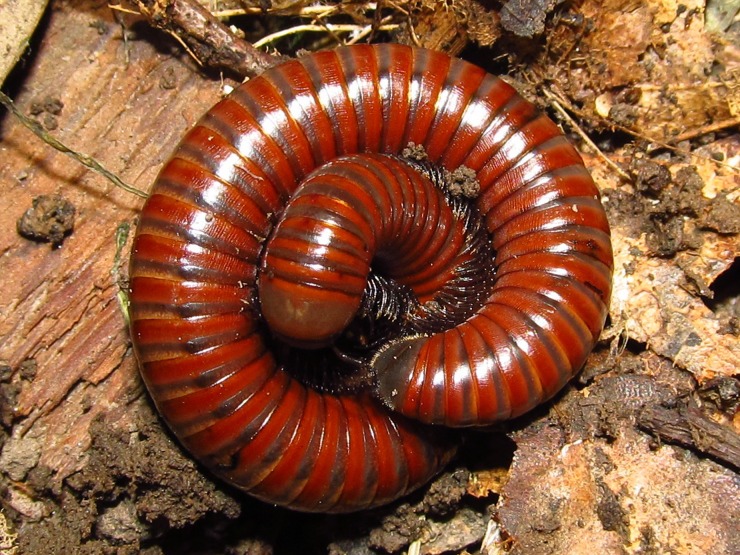
*Pachyiulus hungaricus* (Karsch, 1881) from Mt. Avala, near Belgrade (photo D. Antić).

Aposematic colouration represents the first line of defence in *P*. *hungaricus*, warning predators about unpleasant attributes of its chemical secretion. The second line of defence is the possibility of spiral twisting, thereby protecting the body’s soft parts, primarily the head and anal region (Fig 2). The last line of defence is the use of chemical irritants. When two lines of defence fail, specimens discharge their secretion, first from glands located near the pleurotergites stimulated. *Pachyiulus hungaricus* has the ability to discharge chemicals to the distance of a few centimetres (personal observation). The analysed species has a pair of defensive glands (ozadenes) on each of most of the body’s pleurotergites; only the first five somites and the last one are devoid of glands. The defensive glands in the studied species belong to the juliform type [8].

### Measurement of antimicrobial activity using the well-diffusion method

**Fig 3:**
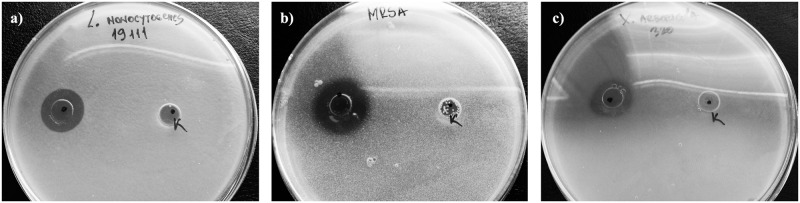
*In* v*itro* effects of the defensive secretion against *L*. *monocytogenes* (a), methicillin-resistant *S*. *aureus* (b) and *X*. *arboricola* (c). K) Negative control of solvent.

In the present study, the defensive secretion of *P*. *hungaricus* was evaluated for its antimicrobial activity against certain Gram-negative and Gram-positive bacteria regarded as human-opportunistic pathogenic microorganisms except in the cases of *X*. *arboricola*, which is a plant pathogen, and *B*. *subtilis*, which was selected as the traditional model system of Gram-positive bacteria. The antibacterial potential of the extract was evaluated according to its zone of inhibition against various pathogens (Fig 3).

The results of initial screening revealed that the extract is a potent antimicrobial agent against all of the microorganisms studied (Table 2). The defensive extract showed the largest inhibition zone (measuring 25 mm in diameter) against two Gram-positive bacteria, *S*. *aureus* and *B*. *subtilis*. The extract exhibited a slightly smaller zone of inhibition (19 mm in diameter) against MRSA and *E*. *coli*. The smallest zone of inhibition was observed against *P*. *aeruginosa*, which proved to be the most resistant strain.

**Table 2:** Antibacterial activity of defensive secret extract from *Pachyiulus hungaricus* (Karcsh, 1881).

Name	ATTC No.	Zone of inhibition (mm) ^b^	MIC (mg/ml)	MBC (mg/ml)	MIC of rifampicin (mg/ml)	MBC of rifampicin (mg/ml)
*Aeromonas hydrophila*	49140	16	0.20	0.40	0.10	0.20
*Pseudomonas aeruginosa*	15442	10	0.60	0.80	0.20	-
*Escherichia coli*	25922	19	0.60	1.00	0.20	0.40
*Xanthomonas arboricola*	IZB 301 ^a^	14	0.40	0.60	0.20	0.40
*Listeria monocytogenes* 1/2a	19111	16	0.20	0.40	0.40	-
*Staphylococcus aureus*	25923	25	0.40	0.60	0.20	0.40
Methicillin resistant *Staphylococcus aureus* (MRSA)	33591	19	0.25	0.50	0.12	0.18
*Bacillus subtilis*	6633	25	0.60	1.00	0.08	0.20

^a^ The strain *X*. *arboricola* was isolated from walnut fruit.

^b^ Mean values of three experiments, expressed as the diameter of the inhibition zone in mm includes the diameter of well (5 mm).

### Determination of MIC and MBC

Determination of the MIC is important in diagnostic laboratories because it helps in confirming the resistance of microorganisms to an antimicrobial agent and in monitoring the activity of new antimicrobial agents [24]. A broth microdilution method was used to determine MIC and MBC values of the defensive secretion, which in the initial screening showed activity against seven bacterial strains. The lowest concentration (highest dilution) of the extract that produced visible bacterial growth on a solid medium but did not change the colour in the selected wells was regarded as MIC. The concentration of extract that completely killed the selected bacteria was taken as MBC. Methanol, which was used as a negative/solvent control, did not have an inhibitory effect on the tested strains. Although spectrometric reading at a specific wavelength is a more objective and quantifiable method, due to hard colouration of the defensive secretion and failure to get correct readings, the resazurin reaction was used instead. Resazurin is an oxidation–reduction indicator used for evaluation of cell growth, particularly in various cytotoxicity assays [25]. It is a purple, non-toxic indicator that turns pink when reduced to fluorescent resorufin by cellular oxidoreductases. The concentration of viable cells in a suspension containing resazurin directly determines the time-point of visible conversion from purple to a pink colour [26].

The results of this study indicated that all strains differ slightly in their sensitivity to the extract. The lowest recorded concentration was 0.20 mg/mL against *A*. *hydrophila* and *L*. *monocytogenes* (Table 2). In the employed range of concentrations, the sensitivity of MRSA was confirmed, although at the slightly higher concentration of 0.25 mg/mL. Moreover, it was noted that for most of the tested strains the antibacterial properties of this extract show MBC values that are one degree of magnitude higher than the corresponding MIC values. The highest bactericidal concentration was 1 mg/mL. The extract of *P*. *hungaricus* was not more effective in inhibiting the growth of pathogenic bacteria than was a commercial antibiotic rifampicin (requiring a one or two times higher concentration than that of the antibiotic), except in the case of *B*. *subtilis*, for whose inhibition a more than seven-fold higher concentration of the extract was required. Only with *L*. *monocytogenes* was the obtained MIC two times lower for the extract than for the antibiotic.

In general, the investigated extract showed that the MIC values for Gram-negative bacteria were slightly higher in comparison to Gram-positive strains except in the case of *A*. *hydrophila*. The structure of Gram-positive bacteria cell wall, with predominant share of peptidoglycan, allows hydrophobic molecules to penetrate the cells and act on wall, as well as cell membrane and within the cytoplasm [27]. The cell wall of Gram-negative bacteria is more complex with less peptidoglycan and with outer membrane composed of double layer of phospholipids linked with inner membrane by lipopolysaccharides [28]. Another possible reason is their possession of multi drug resistance pumps, which extrude amphipathic toxins across the outer membrane [29].

### Determination of MIC and MFC

Based on the obtained results, it can be stated that the defensive secretion of *P*. *hungaricus* exhibited a strong antifungal potential. The growth of phytopathogenic fungi was inhibited at quite similar, lower concentrations. The obtained MIC values ranged from 0.10 to above 0.35 mg/mL, but for most of the tested fungi a concentration of about 0.2 mg/mL was enough to inhibit fungal growth (Table 3).

**Table 3:** Antifungal activities of defensive secret from *Pachyiulus hungaricus* (Karsch, 1881).

Species of fungi	MIC	MFC	MIC of fluconazole	MFC of fluconazole
*Aspergillus flavus*	>0.35	-	1.00	1.60
*Aspergillus niger*	0.20	0.25	1.80	2.20
*Fusarium subglutinans*	0.23	0.25	1.60	2.00
*Fusarium semitectum*	0.20	0.23	1.60	1.80
*Fusarium equiseti*	0.10	0.13	1.00	1.40
*Penicillium* sp.	0.28	0.28	1.80	2.20
*Gliocladium roseum*	0.13	0.13	2.00	2.00
*Chaetomium* sp.	0.18	0.18	1.60	1.80

The values are expressed in mg/ml.

The lowest concentration necessary for inhibition of visible growth was for *Fusarium equiseti* (MIC was 0.10 mg/mL), whereas for *A*. *flavus* a concentration of 0.35 mg/mL was insufficient for complete inhibition of growth. A higher tolerance to this extract was exhibited by *Penicillium* sp., with MIC of 0.28 mg/mL. The values of MFC (the concentration required for complete inhibition of fungal growth) were either the same or slightly higher than the obtained MIC values and ranged from 0.13 mg/mL for *F*. *equiseti* to above 0.35 mg/mL for *A*. *flavus*. The results indicate that fungi were more sensitive to the tested extract than were bacteria, which required higher concentrations (with MICs ranging from 0.20 to 0.60 mg/mL, see above) for inhibition of growth. Also, the extract of *P*. *hungaricus* was more effective in inhibiting the growth of pathogenic fungi than was the commercial antibiotic fluconazole, which inhibited the growth of fungi only at a 10 times higher concentration.

The fungi appear to be more sensitive than bacteria in general. Susceptibility differences between bacteria and fungi may be due to cell wall structural differences between these types of microorganisms. In that sense, the cell wall, especially of Gram-negative bacteria (made up of lipopolysaccharides) is complex and multilayered structure, which makes access to membrane more restricted and barrier to many environmental substances including plant extracts, synthetic and natural antibiotics [30,31]

### General observations

In arthropods, numerous different antibacterial substances have been described and characterized [32]. They differ in regard to their mode of action, activity, tissue of formation and chemical composition. The search for antimicrobials from natural sources as important medical need especially for Gram-negative infections, has received much attention and efforts have been made to identify compounds that can act as suitable antimicrobial agents. This study assessed for the first time the effect of extracts from *P*. *hungaricus* on pathogenic microorganisms. All of what has been said above indicates a strong antimicrobial potential of the tested extracts. It can be postulated that predominant components of the extract ensuring such an antimicrobial potential are 2-methyl-1,4-benzoquinone (**2**) and 2-methoxy-3-methyl-1,4-benzoquinone (**4**), which were probably responsible for the observed effect. Research on quinones of plant origin has shown that simple 1,4-benzoquinones exhibit significant antimicrobial activity against *Staphylococcus epidermidis* (MIC of 0.01 mg/mL) and good activity against the yeasts *Cryptococcus neoformans* (0.08 mg/mL) and *Candida albicans* (0.04 mg/mL) [33]. According to Beheshti et al. [34] compounds containing a quinone group are known to possess various physiological activities, such as antibacterial, antifungal, antiviral, antimicrobial and anticancer. Information concerning the antibacterial and antifungal effects of millipede defense secretions is very scarce. Roncadori et al. [35] analysed the antifungal activity of defensive secretions in four polydesmids, but these species are cyanogenic and produce compounds (benzoic acid, benzoyl cyanide, mandelonitril benzoate, etc.) that are different from those produced by juliform species. Our results can be compared with a study of the defensive secretion in the juliform spirostreptid *Orthoporus antillanus* (Pocock, 1894) [now valid as *Orthoporus cavicollis* (Karsch, 1881)] [36]. In this species, the two dominant compounds (which comprise 96% of the secretion) are 2-methyl-1,4-benzoquinone (**2**) and 2-methoxy-3-methyl-1,4-benzoquinone (**4**). In addition to this, the following four minor quinones were detected as well: 2-hydroxy-3-methyl-1,4-benzoquinone (**3**), 2,3-dimethoxy-1,4-benzoquinone (**8**), 2,3-dimethoxy-5-methyl-1,4-benzoquinone and 2,5-dimethyl-3-methoxy-1,4-benzoquinone. Compounds **2** and **4** are also detected as major constituents of defensive fluids in *P*. *hungaricus*. The secretion of *O*. *cavicollis* was evaluated for its action against three pathogenic human fungi (*Fonsecaea pedrosoi*, *Candida albicans* and *Microsporum gypseum*), the soil nematode *Rotylenchus reniformis*, the human parasitic nematode *Strongyloides stercoralis*, the plant fungus *Fusarium oxysporium* and the bacterium *Xanthomonas campestris*. Results of this study showed that the secretion of *O*. *cavicollis* was toxic to all of the organisms tested. Rocha et al. [37] noted that most species with a quinonic chemoprofile live in the soil, where they are in direct contact with many pathogenic microorganisms. The same authors indicated that MIC values for benzoquinones are low and asserted that these chemicals are very effective at deterring microorganisms. It is important to note that recent studies showed a great variety of Laboulbeniales fungi paraziting millipedes; populations of some julids had especially high infection rate [38,39]. Antifungal potential of the defensive secretion in analyzed species have to provide their better protection against fungal attack.

## Conclusions

This study represents the first report treating the antimicrobial activity of defensive secretions from *P*. *hungaricus*. The investigated extract showed that the MIC values for Gram-negative bacteria were slightly higher in comparison to Gram-positive strains except in the case of *A*. *hydrophila*. These differences are whether probably due to the structure of the cell wall of Gram-negative bacteria which is more complex. In general, the tested extract showed fairly strong antimicrobial activity against both type of bacteria. The defensive secretion also exhibited a stronger antifungal potential. The results indicate that fungi were more sensitive to the tested extract than were bacteria and that the extract was more effective than a commercial antibiotic. The given extract contains antimicrobial components potentially useful as therapeutic agents in the pharmaceutical and agricultural industries.
